# Gut microbiota can transfer fiber characteristics and lipid metabolic profiles of skeletal muscle from pigs to germ-free mice

**DOI:** 10.1038/srep31786

**Published:** 2016-08-22

**Authors:** Honglin Yan, Hui Diao, Yi Xiao, Wenxia Li, Bing Yu, Jun He, Jie Yu, Ping Zheng, Xiangbing Mao, Yuheng Luo, Benhua Zeng, Hong Wei, Daiwen Chen

**Affiliations:** 1 Animal Nutrition Institute, Sichuan Agricultural University, Key Laboratory of Animal Disease-Resistance Nutrition, Ministry of Education, China, Ya’an 625014, People’s Republic of China; 2 Department of Laboratory Animal Science, College of Basic Medical Sciences Third Military Medical University, 30 Gaotanyan Street, Chongqing 400038, China

## Abstract

Obesity causes changes in microbiota composition, and an altered gut microbiota can transfer obesity-associated phenotypes from donors to recipients. Obese Rongchang pigs (RP) exhibited distinct fiber characteristics and lipid metabolic profiles in their muscle compared with lean Yorkshire pigs (YP). However, whether RP have a different gut microbiota than YP and whether there is a relationship between the microbiota and muscle properties are poorly understood. The present study was conducted to test whether the muscle properties can be transferred from pigs to germ-free (GF) mice. High-throughput pyrosequencing confirms the presence of distinct core microbiota between pig breeds, with alterations in taxonomic distribution and modulations in β diversity. RP displayed a significant higher Firmicutes/Bacteroidetes ratio and apparent genera differences compared with YP. Transplanting the porcine microbiota into GF mice replicated the phenotypes of the donors. RP and their GF mouse recipients exhibited a higher body fat mass, a higher slow-contracting fiber proportion, a decreased fiber size and fast IIb fiber percentage, and enhanced lipogenesis in the gastrocnemius muscle. Furthermore, the gut microbiota composition of colonized mice shared high similarity with their donor pigs. Taken together, the gut microbiota of obese pigs intrinsically influences skeletal muscle development and the lipid metabolic profiles.

Specialized microbial communities inhabit a majority of the epithelial surfaces of our body, such as the skin, mucosal surfaces and gastrointestinal tract, with by far the greatest number of bacterial cells in the distal gut^1^. The mammalian distal gut microbiome exceeds the size of the mammalian nuclear genome by two orders of magnitude and has the potential to add a broad range of biological functions that the host could not otherwise perform^2^. This commensal microbiota plays a major role in maintaining human health, providing nutrients, shaping the immune system and modulating gastrointestinal development; indeed, it is sometimes referred to as our “forgotten organ”^3^. The gut microbiota is highly variable from individual to individual and in different body sites in a single host. Environmental and stochastic factors strongly affect the composition of the microbiota, and accumulating evidences indicate that host genotypes and phenotypes influence and interact with the gut microbiota in various mammals^4^,^5^,^6^. In genetically obese mice and obese patients, there are significant differences in the gut microbiota composition compared with lean controls, and these modifications can also be induced by deleting or adding one gene to a model host organism^7^,^8^. In studies of monozygotic or dizygotic twin pairs, considerable differences in the gut microbiota composition were found between healthy co-twins and co-twins with obesity or malnourishment^9^,^10^. These studies strengthened the notion that a host with different phenotypes or genotypes harbors a distinct gut microbiota. The Rongchang pig (RP) is a typical native pig breed from Southwestern China and is characterized by a high body fat mass, excellent meat quality and high intramuscular fat content in the skeletal muscle^11^. The Yorkshire pig (YP) is an imported breed and is characterized by a high body lean mass, an inferior meat quality and low lipid storage in the skeletal muscle^12^. Previous studies also showed that pigs with obese phenotypes have a distinct gut microbiota compared with lean pigs^13^. Thus, we speculated that there are significant differences in the gut microbiota compositions between YP and RP.

Obesity is a major challenge to health care systems worldwide, and is a widely recognized risk factor for various metabolic disorders such as fatty liver and type 2 diabetes. There is substantial evidence suggesting that skeletal muscle properties including fiber characteristics, fiber type distribution and lipid metabolic profile, are closely associated with the presence of obesity^14^,^15^. Interestingly, the gut microbiota is reported to be a causal factor of obesity-associated phenotypes, as the metabolic phenotypes can be transferred from donors to recipients through fecal microbiota transplantation^9^,^16^,^17^. Less attention has been paid to the link between the gut microbiota composition and skeletal muscle development and the metabolic profile. Only a limited number of studies suggest that the depletion of gut microbiota leads to increased muscle fatty acid catabolism^18^. Other accumulating indirect evidence indicates that skeletal muscle development and the metabolic profile are influenced by the ingestion of probiotics/prebiotic^19^,^20^. Such findings make it tempting to speculate that a relationship may exist between muscle properties and the gut microbiota. Whether the skeletal muscle properties are transmissible via fecal microbiota transplantation remains unclear. Our previous study demonstrated that RP exhibit a higher slow-contracting fiber percentage and intramuscular fat content compared to an imported pig breed^21^. Therefore, we hypothesized that the differences in fiber characteristics, fiber type distribution and lipid metabolism in skeletal muscle between pig breeds can be transferred from the pig donors to germ-free (GF) mouse recipients.

The objectives of present study were to investigate differences in the gut microbiota between YP and RP, and to determine whether the differences in the skeletal muscle properties are transmissible via fecal microbiota transplantation. Pigs share high similarity with humans in terms of physiology, organ development and disease progression^22^. Thus, elucidating differences in the gut microbiota between obese pigs and lean pigs and the relationships between the gut microbiota and muscle properties is not only essential for determining the role of the gut microbiota in lipid metabolism and the development of skeletal muscle in pigs but could also reflect the corresponding role of the gut microbiota in humans.

## Results

### Significant differences in the microbiota between pig breeds and microbiota-associated phenotypes can be transferred from pig donors to GF mouse recipients

**Figure 1:**
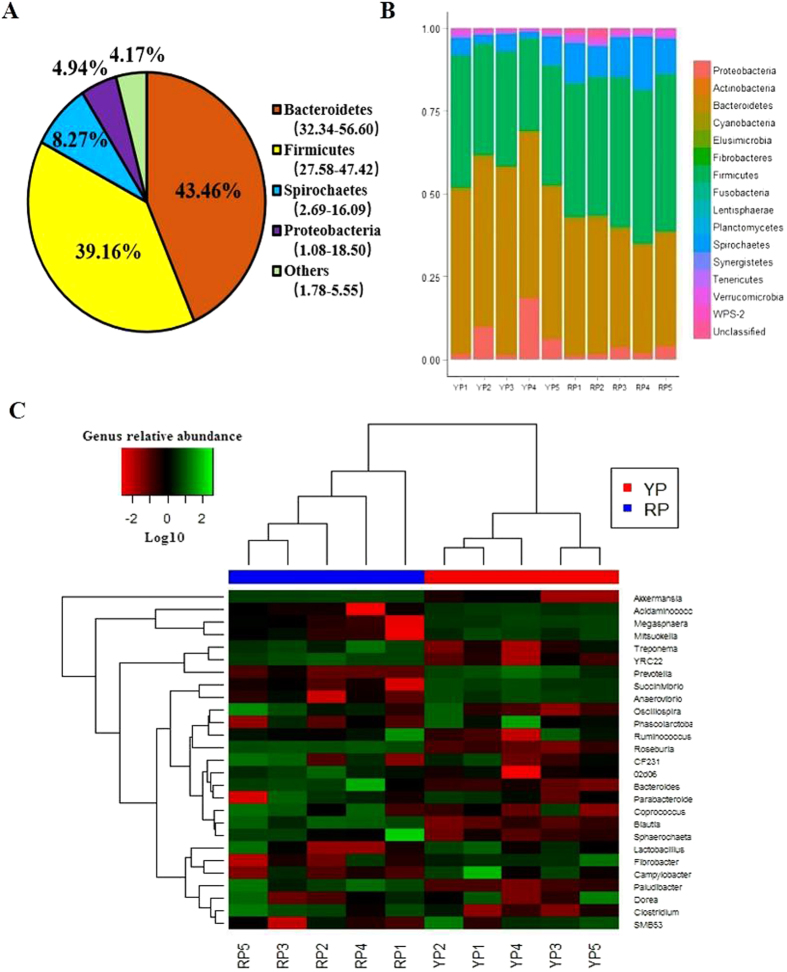
16S rRNA gene analysis reveal phylum- and genus-level differences in the YP and RP microbiota. (**A**) Phylum-level assignments of the assignable 16S rRNA gene sequences from the swine feces, averaged across all 10 individual samples. (**B**) Relative abundance levels of the bacterial phyla present in YP and RP. (**C**) Heatmap of log10-transformed abundance levels of the selected genera (>0.1% in at least one sample) for the individual YP and RP samples. The pigs with the highest and lowest bacterial levels are in green and red, respectively. YP, Yorkshire pigs; RP, Rongchang pigs.

**Figure 2:**
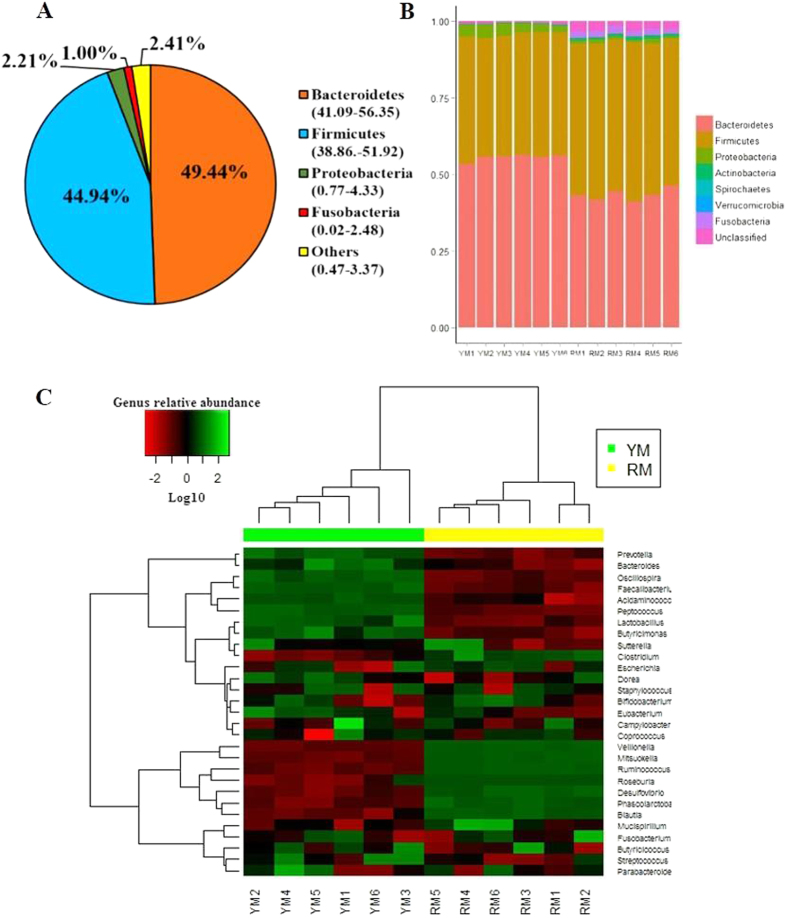
16S rRNA gene analysis reveals phylum- and genus- level differences in YM and RM microbiota. (**A**) Phylum-level assignments of the assignable 16S rRNA gene sequences from the mice feces, averaged across all 12 individual samples. (**B**) Relative abundance levels of the bacterial phyla present in YM and RM. (**C**) Heatmap of log10-transformed abundance levels of all of the observed genera for the individual YM and RM samples. Mice with the highest and lowest bacterial levels are in green and red, respectively. YM, Yorkshire pig fecal microbiota-associated mice; RM, Rongchang pig fecal microbiota-associated mice.

A total of 569,248 high quality sequences were obtained from all pig samples (Table S1), with an average of 56,925 sequences per sample and a range of 32,013 to 69,295. These sequences were assigned to 1,796 operational taxonomic units (OTUs), with 809 of those existing in two pig breeds was identified as the core OTUs (Fig. S1A). The core OTUs represented 58.69% of all of the reads. A total of 10,430,445 high-quality sequences were acquired from 12 mouse samples (Table S1). A total of 4,406 OTUs were generated, with 2,114 of those existing in the two groups identified as the core OTUs (Fig. S2A). The core OTUs represented 51.38% of all of the reads. The fecal samples of all of the pigs were dominated by four phyla: *Bacteroidetes*, *Firmicutes*, *Spirochaetes*, and *Proteobacteria* (Fig. 1A). A total of 15 phyla were shared by the two pig breeds (Fig. 1B). Seven phyla (>1% in at least 1 sample) were chosen for the significance analysis, and an adjusted p value was adopted. Compared with YP, RP had higher proportions of bacteria in *Firmicutes* and *Spirochaetes* and a lower proportion of bacteria in *Bacteroidetes* (p < 0.05) (Fig. S2). All of the mouse fecal samples were dominated by four phyla: *Bacteroidetes*, *Firmicutes*, *Proteobacteria* and *Fusobacteria* (Fig. 2A). The results shown in Fig. 2B described the phylotype distribution at the phyla level for the mouse recipients, and specific microbiota phyla present in the pig donors were also detected in the mouse recipients. The phyla differences were replicated, and a higher proportion of *Firmicutes* and a lower proportion of *Bacteroidetes* were observed in RM versus YM (p < 0.05). In addition, YM exhibited a higher proportion of *Proteobacteria* and a lower proportion of *Fusobacteria* and *Actinobacteria* (p < 0.05) (Fig. S4). Only 58.67% of the total sequences derived from pig fecal samples were assigned to 32 known genera. Ten abundant genera (>1%) were detected in the YP samples, while 9 abundant genera were detected in the RP samples. Fig. 1C presents a heatmap showing the abundances of the selected genera (>0.1% in at least 1 sample) across all of the samples, clearly showing that there are apparent differences in the genus distribution between YP and RP fecal microbiota. The proportions of *Treponema*, *YRC22*, *Oscillospira*, *Roseburia*, *Ruminococcus*, *Paludibacter*, *Coprococcus* and *Blautia* were higher in RP, whereas the proportions of bacteria in *Prevotella*, *Succinivibrio*, *Anaerovibrio*, *Lactobacillus*, *Bacteroides*, *Acidaminococcus*, *Megasphaera* and *Mitsuokella* were higher in YP. Likewise, the bacterial genera distribution differed between Yorkshire pig flora-associated mice (YM) and Rongchang pig flora-associated mice (RM), and several genera differences existing in the pig donors were conserved in the mouse recipients. *Roseburia*, *Ruminococcus* and *Blautia* were more and *Prevotella* was less represented in RM compared to YM (Fig. 2C). For the α and β diversity analyses, the sequence number for each sample from both pigs and mice was rarefied to 30,000 by randomly subsampling to minimize the variation in sequencing depth among the samples. There were no significant differences in the observed OTUs and the Chao 1 index between the YP and RP samples (p > 0.05) (Fig. S1B,C). As indicated in the pig donors, there were also no significant differences observed for the OTUs and the Chao 1 index between the mouse recipients (p > 0.05) (Fig. S3B,C).

**Figure 3:**
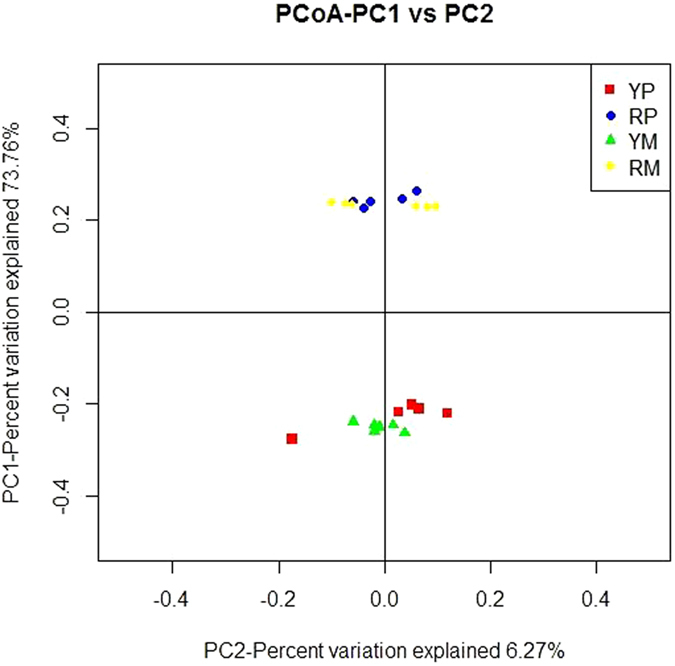
Comparison of the gut microbiota compositions among the 4 groups. A PCoA was used to visualize the weighted UniFrac distances of the fecal samples from the individual pigs and mice. YP, Yorkshire pigs; RP, Rongchang pigs; YM, Yorkshire pig fecal microbiota-associated mice; RM, Rongchang pig fecal microbiota-associated mice.

To measure the extent of the similarity between microbiota communities, a principal coordinate analysis (PCoA) based on weighted UniFrac distance metrics was performed. The fecal microbiota from YP and RP divided into two different clusters that separated clearly in the PCoA (Fig. 3). The community structures observed in the YM samples were significantly different from the communities detected in the RM samples (Fig. 3). The PCoA plots showed that YM and RM samples formed two different clusters, and that, for each condition, the fecal samples of the mouse recipients formed a cluster that was close to its donor fecal samples. Therefore, overall, the bacterial microbiota showed a marked divergence between YP and RP, and the mouse recipients shared high similarity with their pig donors in their gut microbiota.

### The two groups of mouse recipients developed different body compositions, fiber characteristics and fiber type distributions

**Figure 4:**
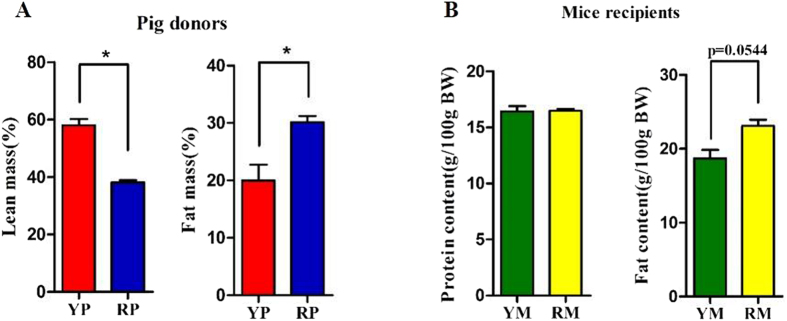
Comparison of the body compositions in pig donors (**A**) and mouse recipients (**B**). YP, Yorkshire pigs; RP, Rongchang pigs; YM, Yorkshire pig fecal microbiota-associated mice; RM, Rongchang pig fecal microbiota-associated mice. *P < 0.05.

**Figure 5:**
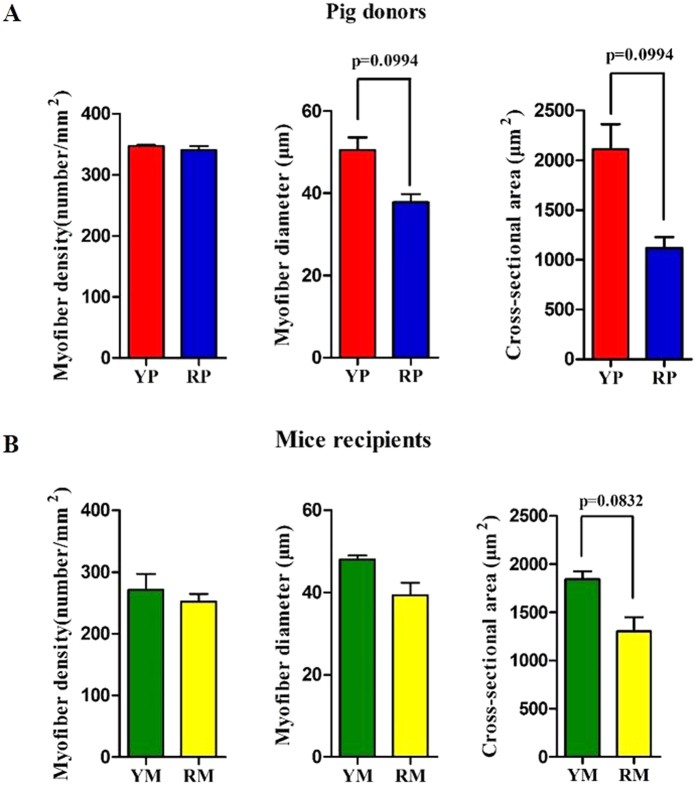
Comparison of the muscle fiber characteristics in the gastrocnemius muscles of the pig donors (**A**) and mouse recipients (**B**). YP, Yorkshire pigs; RP, Rongchang pigs; YM, Yorkshire pig fecal microbiota-associated mice; RM, Rongchang pig fecal microbiota-associated mice. *P < 0.05.

**Figure 6:**
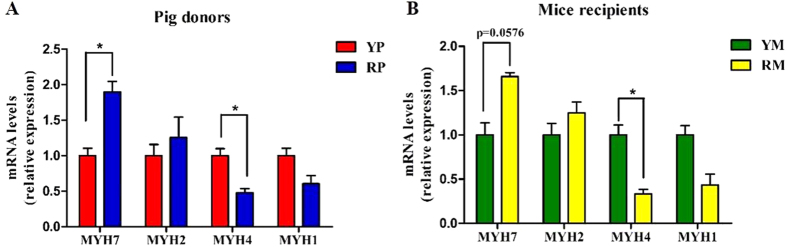
mRNA expression levels of the myosin heavy-chain (MyHC) isoforms genes in the gastrocnemius muscles of the pig donors (**A**) and mouse recipients (**B**). YP, Yorkshire pigs; RP, Rongchang pigs; YM, Yorkshire pig fecal microbiota-associated mice; RM, Rongchang pig fecal microbiota-associated mice. MYH, Myosin heavy chain. *P < 0.05.

Two groups of GF BALB/C mice were colonized with the fecal suspensions prepared from YP and RP (conventionalization). The two groups of mouse recipients were then named Yorkshire pig flora-associated mice (YM) and Rongchang pig flora-associated mice (RM). Consistent with our previous study^21^, obese RP had a higher fat mass and a lower lean mass than lean YP (p < 0.05) (Fig. 4A). Consistently, there was a trend toward a higher body fat mass in RM compared to YM (p = 0.0544) (Fig. 4B). Additionally, the fiber diameter and the cross-sectional area (CSA) of the gastrocnemius muscle (GM) trended toward an increase in the lean YP (p = 0.0994) (Fig. 5A). Likewise, there was a trend toward a larger CSA in the GM of YM (p = 0.0832) (Fig. 5B). Owing to the critical role of the fiber type profile in determining the fiber characteristics, the gene expression levels of myosin heavy-chain (MYHC) isoforms in the GM of the pig donors and mouse recipients were measured. As expected, MYH7, which encodes the slow-contracting fiber, was expressed at higher levels in the GM of the RP donors and their GF recipients (p < 0.05). Additionally, the expression of MYH4, which encodes the fast IIb MYHC, was reduced in the GM of RP and RM (p < 0.05) (Fig. 6A,B).

### Obese pig-derived microbiota enhances lipogenesis in skeletal muscle

**Figure 7:**
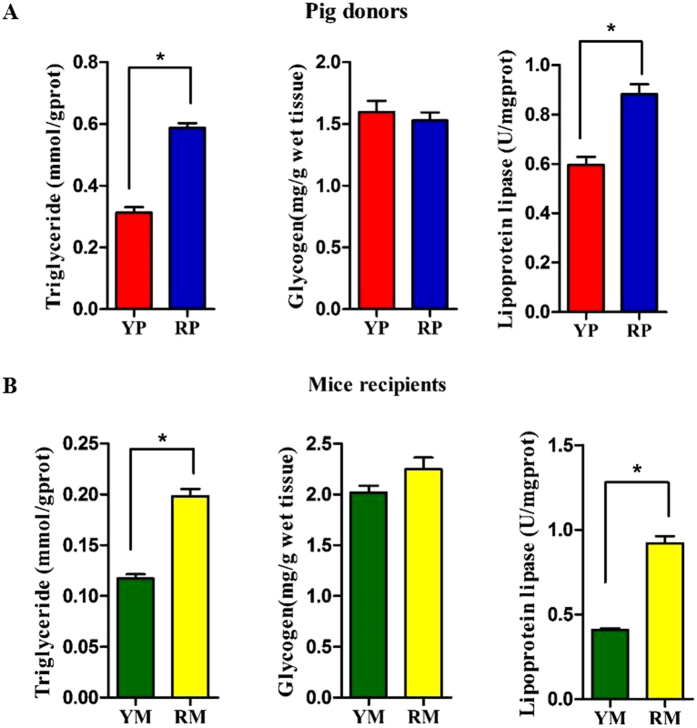
Differences in the metabolites content and the lipoprotein lipase activity in the gastrocnemius muscles of the pig donors (**A**) and mouse recipients (**B**). YP, Yorkshire pigs; RP, Rongchang pigs; YM, Yorkshire pig fecal microbiota-associated mice; RM, Rongchang pig fecal microbiota-associated mice. *P < 0.05.

**Figure 8:**
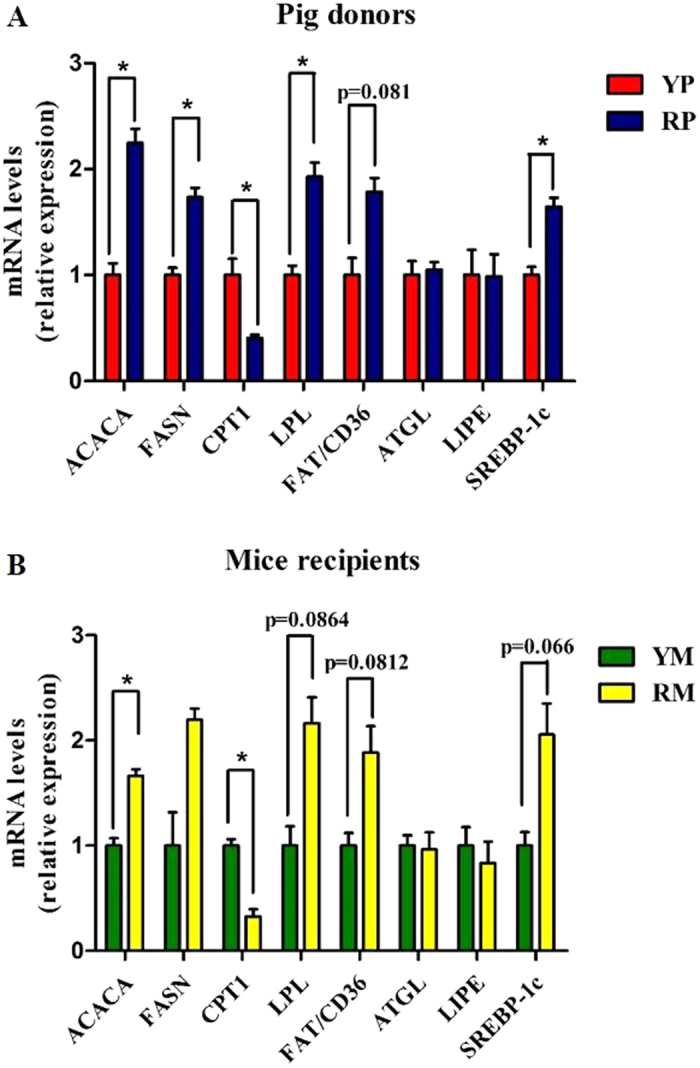
mRNA expression levels of the lipid metabolism-related genes in the gastrocnemius muscles of the pig donors (**A**) and mouse recipients (**B**). YP, Yorkshire pigs; RP, Rongchang pigs; YM, Yorkshire pig fecal microbiota-associated mice; RM, Rongchang pig fecal microbiota-associated mice. ACACA, acetyl-CoA carboxylase alpha; FASN, Fatty acid synthase; CPT1, carnitine palmitoyl transferase 1; LPL, Lipoprotein lipase; FAT/CD36, fatty acid translocase/CD36; ATGL, Adipose triglyceride lipase; LIPE, Lipase, hormone sensitive; SREBP-1c, Sterol regulatory element binding protein-1c. *P < 0.05.

The gut microbiota alters the expression of host genes involved in lipid metabolism^23^. In the GM, obese RP and their mouse recipients exhibited higher triglyceride (TG) concentrations and lipoprotein lipase (LPL) activity, indicating enhanced lipid deposition (p < 0.05) (Fig. 7A,B). We further analyzed the expression of genes regulating lipid uptake, lipogenesis and lipolysis in the GM. RP exhibited increased expression of acetyl-CoA carboxylase (ACACA) and fatty acid synthase (FASN), but only ACACA abundance was upregulated in RM (p < 0.05). Furthermore, carnitine palmitoyltransferase 1 (CPT1), a transport protein that regulates fatty acid β-oxidation, was reduced in the RP donors and the RM recipients (p < 0.05). Additionally, LPL, which provides fatty acid for tissue utilization and storage, was increased in the RP donors (p < 0.05). There was also a trend towards higher LPL abundance in the RM recipients (p = 0.0864). Moreover, fatty acid translocase/CD36 (FAT/CD36), which imports lipids and lipoproteins, trended toward a higher expression in the RP donors (p = 0.081) and their GF recipients (p = 0.0812). In addition, sterol regulatory element-binding protein-1c (SREBP-1c), a key transcription factor of lipogenesis in skeletal muscle and an activator of ACACA and FASN, was more highly expressed in RP than in YP (p < 0.05). Furthermore, SREBP-1c trended toward increased expression in the RM recipients (p = 0.066). In addition, there were no differences in the expression levels of adipose triglyceride lipase (ATGL) and hormone sensitive lipase (LIPE) between YP and RP or between YM and RM (p > 0.05) (Fig. 8A,B).

## Discussion

The gut microbiota is recognized as a strong determinant of host physiology, especially its critical role in host metabolism^24^. The causal relationship between the gut microbiota and obesity-associated phenotypes has been extensively studied. Obesity-associated phenotypes are transmissible via fecal microbiota transplantation^17^,^25^. There is a wealth of data indicating that the skeletal muscle metabolic profile and the fiber type differed between hosts with metabolic syndrome and healthy individuals^26^. Some authors suggest that a gut microbiota-muscle axis might exist^27^. However, there is not enough evidence to indicate a relationship between the gut microbiota and skeletal muscle. Here, we demonstrated that obese RP exhibit distinct and different gut microbial communities compared to lean YP. Furthermore, we showed that the transfer of a porcine microbiota replicates the skeletal muscle properties of the pig donors.

Several factors affect the evolution of the mammalian gut microbiota, and host genotypes and phenotypes are considered as main factors contributing to the diversity of the gut microbiota^4^,^28^. Consistent with a recent study^29^, we identified a gut microbiota dominated mainly by *Bacteroidetes*, *Firmicutes*, *Spirochaetes*, and *Proteobacteria* in YP and RP. Studies comparing the gut microbiota between obese and lean animals showed that higher *Firmicutes* and lower *Bacteroidetes* levels were associated with obesity^13^,^30^,^31^. We observed similar results in RP and their mouse recipients. In addition, higher levels of S*pirochaetes* were observed in RP. In the rumen and the termites guts, *Spirochaetes* is capable of degrading polymers commonly present in plant materials. Certain polymers including xylan, pectin and arabinogalactan were fermentable substrates of *Spirochaetes*^32^,^33^. More recently, *Spirochaetes* was determined to be positively correlated with the apparent hemicellulose digestibility of pigs^34^. RP are recognized to be more adaptable to poor dietary conditions than foreign pig breeds^35^, a fact that may be attributed to the higher *Spirochaetes* proportion in the gut microbiota. YP and RP are typically lean and obese pigs^11^,^12^,^36^, and RP and their GF mouse recipients exhibited higher body fat masses in the present study. Studies in humans and rodents showed that differences at the genus level play a great role in obesity^30^,^37^. Most species of *Ruminococcus* belong to the *Clostridium cluster IV*, which has been associated with both obesity and weight loss^38^,^39^. Higher levels of *Ruminococcus* were observed in RP and RM, which is similar to what was observed in obese humans and HF-fed mice^40^,^41^. Species from *Roseburia* and *Blautia* are major bacteria that produce butyrate and acetic acid, respectively^42^,^43^. We found that RP and RM exhibited increased abundance levels of *Roseburia* and *Blautia*, which was supported by previous studies showing an elevation in total short chain fatty acid production in obese individual^30^. *Prevotella* has been recently reported relative to body weight loss in overweight adolescents^38^ and was decreased in RP and RM. Previous studies indicated that the all obese gut communities were more similar to each other than to lean gut communities^16^,^31^, and we also found that YP and RP fecal microbiota could be divided into two separate clusters based on a beta diversity analysis. The recipient mice shared high similarity in bacterial community structure with their corresponding pig donors, which was consistent with previous studies showing that human and rat microbiota can be transferred to GF mice with striking preservation of structure and diversity^37^,^44^. Overall, these data indicated that the gut composition differs between obese RP and lean YP, and the microbiota-related phenotypes of pig donors were preserved and transferred to their GF mouse recipients.

In humans, skeletal muscle represents approximately 40% of the body weight and constitutes the largest organ in our body^45^. During the past decade, it has been increasingly identified that skeletal muscle development and metabolism are closely associated with obesity^46^. There are differences in the fiber type proportions between lean and obese animals^21^,^47^. In the present study, we found that RP and RM exhibited higher amounts of type I fibers and lower amounts of type IIb fibers, consistent with our previous study^21^. Recent studies demonstrated that muscle hypertrophy, owing to increased type IIb fibers, plays an important role in combating diet-induced obesity and metabolic dysfunction^48^,^49^. In agreement, muscle fiber diameter and CSA were lower in RP, and a lower CSA was detected in RM. Obesity leads not only to an increased lipid deposition in adipose tissues but also to the infiltration of fat in other tissues, such as the liver and skeletal muscle^50^. We also found that obese pig donors and their mouse recipients had higher TG content in the GM. Previous studies indicated that lipid storage in non-adipose tissue attributed to an increased uptake of free fatty acid together with a reduced fatty acid catabolism in those tissues^51^. In this regard, RP and RM exhibited elevated LPL and FAT/CD36 mRNA levels in the GM, indicative of increased fatty acid uptake into skeletal muscle^52^. Higher ACACA and SREBP-1c and lower CPT-1 mRNA abundance levels were observed in the GM of obese pig donors and RM, indicating that the obese state enhances lipogenesis and inhibits fatty acid catabolism in skeletal muscle^51^. Collectively, these results indicate that obese state-induced alterations to the gut microbiota affect muscle properties including fiber characteristics, fiber type distribution and lipid metabolism. While previous studies have shown that fecal microbiota transplantations into GF mouse recipients can replicate several aspects of the obesity-associated phenotypes^16^,^17^, this is the first demonstration that skeletal muscle fiber proportions and lipid metabolic profiles can be transferred from pig donors to mouse recipients. Our study provides new evidences supporting the existence of a gut microbiota-muscle axis^27^.

In conclusion, this study demonstrates the large distinction in the gut microbiota composition between obese and lean pigs and reveals the contribution of the gut microbiota to the regulation of fiber characteristics, fiber type distribution and lipid metabolism in skeletal muscle. Taken together, these data demonstrate that obese individuals may harbor a specific gut microbiome that enhances ectopic fat deposition in skeletal muscle and inhibits muscle growth. These findings provide new approaches to intervene host metabolism and animal phenotypes.

## Methods

### Animal husbandry

All of the experimental procedures and animal care were performed in accordance with the Guide for the Care and Use of Laboratory Animals prepared by the Institutional Animal Care and Use Committee of Sichuan Agricultural University, and all of the animal protocols were approved by the Animal Care and Use Committee of Sichuan Agricultural University under permit number DKY-B20131704.

Pigs: Rongchang pigs (RP; n = 5) and Yorkshire pigs (YP; n = 5) were provided by a reservation farm. YP and RP were housed separately in two environmentally controlled room that allowed ad libitum access to water and diet. Starting at 12 weeks of age, the pigs were fed a regular diet for 8 weeks, until euthanasia. The diet (Table S2) was formulated to meet or exceed current NRC (2012) recommendations for all nutrients for 25- to 50-kg pigs. There was no significant difference in the feed intake between pig breeds (data not shown).

Mice: A total of 20 1-day-old germ-free (GF) BALB/C mice were provided by the Department of Laboratory Animal Science of the Third Military Medical University and were used as recipients for the fecal microbiota transplantation. The GF mice were housed in sterile plastic film isolators and were given ad libitum access to sterilized water during the whole course of the experiment. One-day-old mice were breast fed by the GF foster mice until weaning (weaned at 3 weeks of age) and were then fed ad libitum with a sterilized chow diet for 2 weeks post-weaning.

### Fecal microbiota transplantation

According to the criteria for donor identification and screening described by Hamilton *et al.*^53^, the pigs used in the current study consumed a regular diet without antibiotics and probiotics for 8 weeks prior to feces collection. Spontaneously excreted feces were collected from all of the pigs during the final week prior to the slaughter. To acquire representative fecal material for each breed, parts of the fecal samples derived from pigs within the same breed were mixed and were then used as the fecal inoculum. The remaining feces derived from the pigs were stored at −80 °C until DNA extraction. The stool suspension was prepared as we previously described^54^. Newborn GF mice (n = 10 for each pig breed) were colonized with 0.05 ml of the porcine fecal suspension using a nasogastric tube, and 2-ml aliquot suspensions were spread on the fur of each foster mouse. Two treatments were produced: Rongchang porcine flora-associated mice (RM) and Yorkshire porcine flora-associated mice (YM).

### Sample collection from the donors and recipients

Spontaneously excreted fecal samples were collected from 12 male mice (6 mice per treatment) in the last two days prior to euthanasia and were immediately stored at −80 °C until DNA extraction. All of the pigs were euthanized at 20 weeks of age as we previously described^55^, and all of the mice were euthanized at 5 weeks of age. The GM from the pigs (n = 5 per breed) and mice (n = 6 per group) was collected at euthanasia for further histological and molecular analyses. The remaining intact mice (n = 4 per group) were only used for body composition analyses.

### DNA extraction and microbiota analysis

Total DNA was isolated and purified using the QIAamp DNA stool Mini Kit (Qiagen, GmbH Hilden, Germany) modified to contain a bead-beating step. The concentration and purity of the extracted genomic DNA were measured using a NanoDrop ND-1000 spectrophotometer (NanoDrop, Germany). The integrity of the extracted genomic DNA was determined by electrophoresis on a 1% (w/v) agarose gel.

Sequencing and bioinformatics analysis were performed by BGI (Shenzhen, China). Prior to high-throughput sequencing, a DNA library was prepared as previously described^56^. Briefly, the DNA extracted from the fecal samples was used as a template to amplify the hypervariable regions V3 and V4 of 16S ribosome RNA gene. The primers contained base pair sequence complementary for the V3 and V4 regions and illumine adaptors and molecular barcodes as previously described^57^. The resulting amplicons were gel purified, quantified, pooled and sequenced using the 250-bp paired-end reads strategy on the Illumina HiSeq 2000 platform.

The resulting sequences were clustered into OTUs using USEARCH drive5 at 97% sequence similarity. The chimeric OTUs were removed using UCHIME v4.2. Representative sequences for each OTU were picked and aligned using QIIME 1.8. The Ribosomal Database Project classifier v2.2 was used to assign a taxonomic rank to each sequence in the representative set. The relative abundance of each OTU was examined at different taxonomic levels. To minimize biases caused by sequencing depth, the number of reads per sample (all pig and mouse samples) was randomly subsampled to 30,000. The alpha and beta diversity calculations and the taxonomic community assessments were performed using QIIME 1.8 scripts.

### Carcass compositions of the pigs and body compositions of the mice

To determine the carcass compositions of the pigs, the right side of each carcass was dissected into 4 parts as follows: lean; fat; bone and skin. The lean and fat percentages of the carcass were calculated based on the formula (NY/T 825–2004, Ministry of Agriculture of the People’s Republic of China, 2004) as follows:

Lean percentage (%) = lean weight (kg)/[lean weight (kg) + fat weight (kg) + bone weight (kg) + skin weight (kg)] × 100%.

Fat percentage (%) = fat weight (kg)/[lean weight (kg) + fat weight (kg) + bone weight (kg) + skin weight (kg)] × 100%.

Four mice in each group were chosen for analyzing body compositions. Intact mice were chopped and dried by lyophilization. The dried material was ground to determine the chemical composition of the whole body. Total nitrogen was analyzed using the Kjeldahl method^58^. The fat content of each intact mouse was measured by extraction with petrol ether overnight using a Soxhlet apparatus.

### Histological analyses

The GMs, removed from pigs (n = 5) and mice (n = 6), were fixed in a 10% formalin solution, dehydrated and embedded in paraffin wax. Muscle sections were cut to a thickness of 10 μm and were stained with hematoxylin and eosin to observe the morphology of the muscle tissues. Five sections of each sample were photographed using a digital microscope camera (JVC, Yokohama, Japan) linked with a light microscope (Olympus, Tokyo, Japan) at 40X magnification. Image-processing software (Image-Pro Plus 4.5, Silver Spring, MD, USA) was used to score all of the parameters in the sections. A total of 500 fibers from five random fields were measured to calculate the mean values of the fiber diameter and the cross-sectional area. As an indicator of fiber density, the number of fibers was also expressed per mm^2^ in the GMs. All of the observations were determined by a single experimenter who was blinded to the pig breed and the source of the gut microbiota.

### Muscle metabolites concentrations and enzyme activity

The glycogen content of the muscle was measured according to the method previously described^59^. Briefly, powdered muscle (50 mg) was hydrolyzed in 150 μl of 1 mol/L KOH by heating at 95 °C for 20 min. The samples were incubated at 37 °C for 2 h, centrifuged at 4 °C/16000 × *g* for 10 min, and neutralized with NaOH. The resulting free glycosyl units were determined using D-glucose as a standard, and the results are expressed as mg/g muscle tissue. The LPL activity and TG content in the skeletal muscle were measured using a Triglyceride Assay Kit and a Lipoprotein Lipase Assay Kit provided by the Nanjing Jiancheng Bioengineering Institute, China. Approximately 100 mg of frozen muscle was minced, weighed and thoroughly homogenized with 1 ml of ice-cold PBS using a mechanical tissue disrupter. After centrifugation (2500 × *g* at 4 °C), the supernatants were decanted and saved for assaying LPL activity, and the TG concentration was measured in triplicate at the appropriate dilutions. The LPL activity is expressed as U/mg protein of muscle tissue, and the TG content is expressed as mmol/g protein of muscle tissue. The total protein content of the supernatant was measured with the Coomassie Brilliant Blue Protein Assay Kit (Nanjing Jiancheng Institute of Bioengineering, China).

### Real-time PCR

Total RNA from the GM was extracted with TRIzol reagent (TaKaRa, Dalian, China) according to manufacturer’s instructions. cDNA was synthesized via reverse transcription, which was performed with 2 μg of total RNA using a PrimeScript RT reagent kit with gDNA Eraser (TaKaRa, Dalian, China). Primers used for the target genes (MYH7, MYH2, MYH4, MYH1, ACACA, FASN, CPT1, LPL, FAT/CD36 ATGL, LIPE and SREBP-1c) were designed using Primer3Plus (Applied Biosystems) based on the certain exon-exon boundaries of published gene sequences of pigs or mice. Quantitative real-time PCR was performed on an ABI Prism 7000 detection system in a two-step protocol with SYBR Green (Applied Biosystems, Foster City, CA, USA). Each 10 μl volume reaction contained 1 μl of cDNA, 5 μl of SYBR Premix Ex Taq TM (2×), 0.2 μl of ROX reference dye (50×), 0.4 μl of each forward and reverse primer, and 3 μl of PCR-grade water. The thermal cycling program included a 1-min pre-incubation at 95 °C, followed by 40 cycles of denaturation at 95 °C for 5 s, a 60 °C annealing step for 25 s, and an extension at 72 °C for 15 s. The expression of the 18S RNA gene for each species was used as an internal control. All of the experimental sample analyses were run in triplicate. The gene expression data were calculated using the 2^−ΔΔCt^ method and are expressed as the ratio of the expression of targeted genes to the 18S RNA housekeeping gene^60^. The relative expression of targeted genes in porcine or murine muscle were normalized to the YP or YM group, respectively.

### Statistical analysis

For all of the parameters, the data were tested for significance with the two-sample *t*-test method using statistic software SAS 9.1 statistic software (SAS Institute Inc., NC, USA) and are expressed as the mean ± SE. To correct for multiple comparisons in the statistical testing, a Bonferroni correction was used to adjust all of the p values. The differences were considered significant when the p values < 0.05. The beta diversity and PCoA plots were produced using weighted UniFrac metrics. The plots were visualized using R software (Package ape). For the heatmap representations, log10-transformation was applied on the genus relative abundance data matrix, which allowed for the visualization of similarities or differences between the samples that affect members of the community that may make up less than 1% of the relative abundance in a sample.

## Additional Information

**How to cite this article**: Yan, H. *et al.* Gut microbiota can transfer fiber characteristics and lipid metabolic profiles of skeletal muscle from pigs to germ-free mice. *Sci. Rep.* **6**, 31786; doi: 10.1038/srep31786 (2016).

## Supplementary Material

Supplementary Information
