# Supplementary material for: Gut microbiota can transfer fiber characteristics and lipid metabolic profiles of skeletal muscle from pigs to germ-free mice
Source: Sci Rep. 2016 Aug 22;6:31786. doi: 10.1038/srep31786 (PMC4992887; doi:10.1038/srep31786)
Supplement: Supplementary Information [file srep31786-s1.pdf]

Gut microbiota can transfer fiber characteristics and lipid metabolic profile of skeletal muscle from pigs to germ free mice

Honglin Yan[1a], Hui Diao[1a], Yi Xiao[1a], Wenxia Li[1b], Bing Yu[a], Jun He[a], Jie Yu[a], Ping Zheng[a], Xiangbing Mao[a], Yuheng Luo[a], Benhua Zeng[b], Hong Wei[b*], Daiwen Chen[a*]

[a]Animal Nutrition Institute, Sichuan Agricultural University, Key Laboratory of Animal Disease-Resistance Nutrition, Ministry of Education, China, Ya'an 625014, People's Republic of China

[b]Department of Laboratory Animal Science, College of Basic Medical Sciences Third Military Medical University, 30 Gaotanyan Street, Chongqing 400038, China

[1]Honglin Yan, Hui Diao, Yi Xiao and Wenxia Li contributed equally to this work.

*Correspondence to: Professor Daiwen Chen, Animal Nutrition Institute, Sichuan Agricultural University, Xinkang Road 46#, Ya'an, Sichuan Province, People's Republic of China, 625014; dwchen@sicau.edu.cn; or Professor Hong Wei, Department of Laboratory Animal Science, College of Basic Medical Sciences Third Military Medical University, 30 Gaotanyan Street, Chongqing 400038, China; weihong63528@163.com.

Supporting information

Table S1 Raw reads, sequences and OTUs among four groups

Group	Raw reads	High quality sequences	OTUs
YP	289739	281318	1224
RP	294033	287930	1381
YM	5521883	5138823	3138
RM	5538960	5291622	3382

YP, Yorkshire pigs; RP, Rongchang pigs. YM, Yorkshire pig fecal microbiota-associated mice; RM, Rongchang pig fecal microbiota-associated mice; OTUs, operational taxonomic units.

Table S2 Feed ingredients and nutrient content of diet

Ingredients, %	content	Nutrient level [a]	content
Corn	78.20	Digestible energy, Mcal/kg	3.40
Soybean meal	14.60	Crude protein, %	15.74
Soybean oil	1.00	Ca, %	0.52
Fish meal	4.50	Total P, %	0.50
Limestone	0.35	Available P, %	0.32
Dicalcium phosphate	0.27	Digestible lysine, %	0.98
Salt	0.25	Digestible Met, %	0.35
L-Lys·HCl	0.34	Digestible Met + Cys, %	0.55
DL-Met	0.10	Digestible Thr, %	0.59
Thr（98.5%）	0.07	Digestible Trp, %	0.17
Trp(98.00%)	0.02		
Chloride choline	0.05		
Vitamin and mineral premix [b]	0.20		

[a] All data were calculated values.

[b] Provided the following (mg/kg diet): Fe, 120 mg; Cu, 17 mg; Zn, 120 mg; Mn, 25 mg; Se, 0.20 mg; I, 0.30 mg; vitamin A, 5512 IU; vitamin D_3, 2250 IU; vitamin E, 24 IU; vitamin K_3, 3 IU; vitamin B_{12}, 0.02 mg; folic acid, 1.20 mg; riboflavin, 6 mg; nicotinic acid, 20 mg; pantothenic acid, 15 mg; biotin, 0.15 mg.

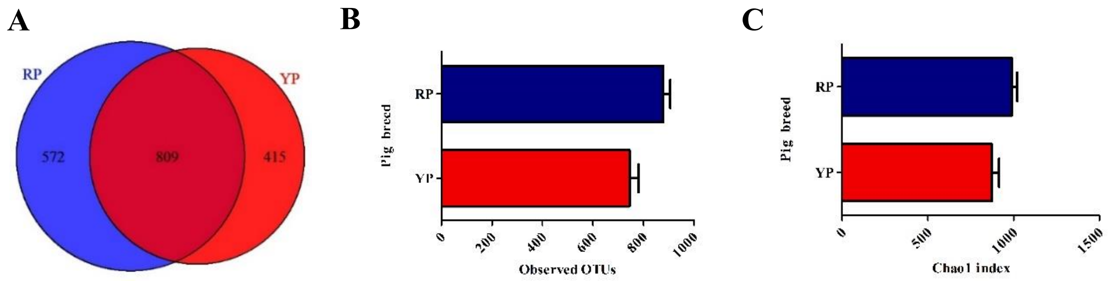

Fig. S1 comparison of the OTUs in the two pig breed. The number of observed OTUs sharing ≥ 97% sequence similarity. (A) A Venn diagram was generated to describe the common and unique OTUs between the two pig breed. (B) From the numbers of OTUs in the two pig breed, there was no difference between YP and RP. (C) The bacterial abundance is reflected in Chao1 index; there was no difference in Chao1 between pig breeds. YP, Yorkshire pigs; RP, Rongchang pigs.

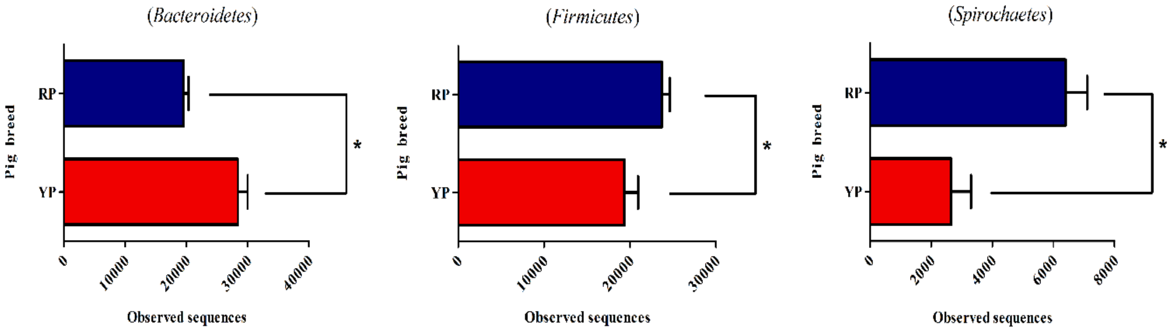

Fig. S2 The bacterial abundances of 3 distinct phyla significantly differ between the two pig breeds. YP, Yorkshire pigs; RP, Rongchang pigs. * $P<0.05$.

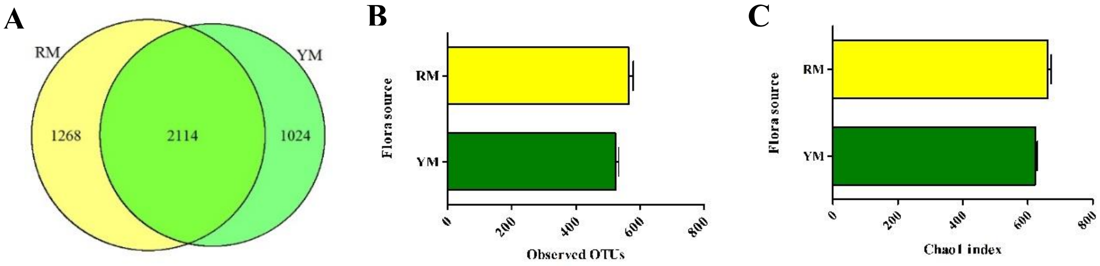

Figure S3. comparison of the OTUs between YP flora-associated mice (YM) and RP flora-associated mice (RM). The number of observed OTUs sharing ≥ 97% sequence similarity. (A) A Venn diagram was generated to describe the common and unique OTUs between YM and RM. (B) From the numbers of OTUs in the two groups, there was no difference between YM and RM. (C) The bacterial abundance is reflected in Chao1 index; there was no difference in Chao1 between mice recipients.

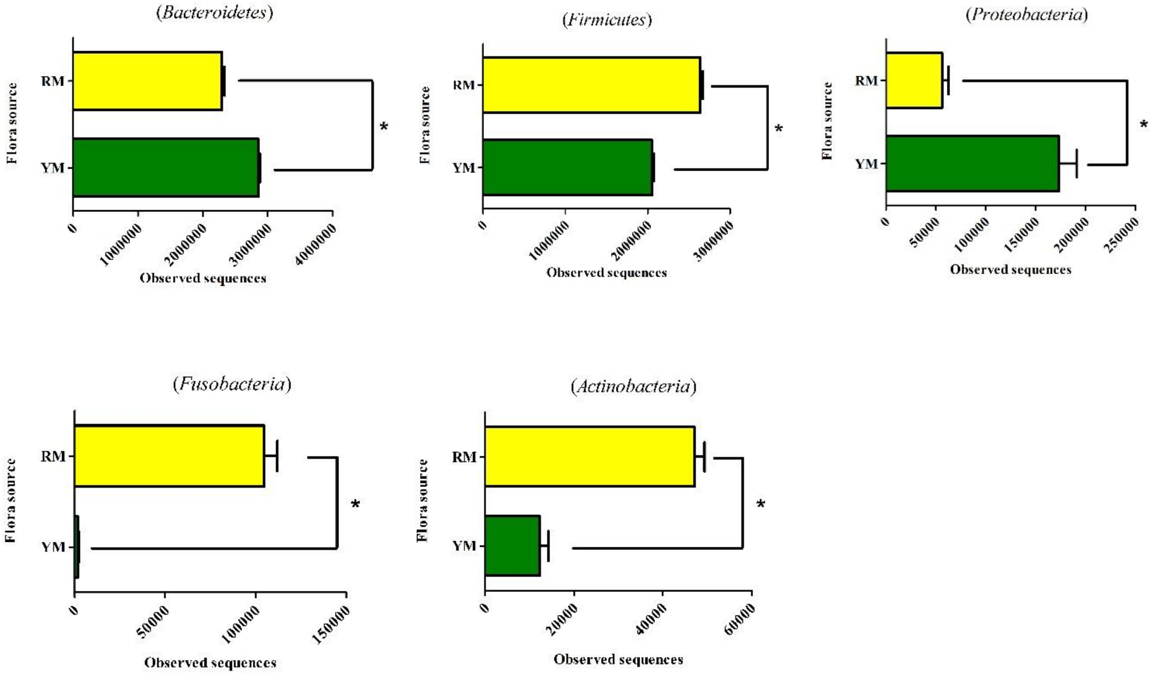

Figure S4. The bacterial abundances of 5 distinct phyla significantly differ between mice recipients. YM, YP flora-associated mice; RM, RP flora-associated mice. * P<0.05.
